# Developmental exposure to methylmercury and ADHD, a literature review of epigenetic studies

**DOI:** 10.1093/eep/dvab014

**Published:** 2021-11-22

**Authors:** Tao Ke, Alexey A Tinkov, Antoly V Skalny, Aaron B Bowman, Joao B T Rocha, Abel Santamaria, Michael Aschner

**Affiliations:** Department of Molecular Pharmacology, Albert Einstein College of Medicine, 1300 Morris Park Avenue, Forchheimer Building, Room 209, Bronx, NY 10461, USA; World-Class Research Center “Digital Biodesign and Personalized Healthcare”, IM Sechenov First Moscow State Medical University (Sechenov University), Moscow 119435, Russia; Laboratory of Ecobiomonitoring and Quality Control, Yaroslavl State University, Yaroslavl 150003, Russia; World-Class Research Center “Digital Biodesign and Personalized Healthcare”, IM Sechenov First Moscow State Medical University (Sechenov University), Moscow 119435, Russia; Laboratory of Medical Elementology, K.G. Razumovsky Moscow State University of Technologies and Management, Moscow 109004, Russia; School of Health Sciences, Purdue University, West Lafayette, IN 47907-2051, USA; Department of Biochemistry and Molecular Biology, Federal University of Santa Maria, Santa Maria, RS 97105-900, Brazil; Laboratorio de Aminoácidos Excitadores, Instituto Nacional de Neurología y Neurocirugía, Mexico City 14269, Mexico; Department of Molecular Pharmacology, Albert Einstein College of Medicine, 1300 Morris Park Avenue, Forchheimer Building, Room 209, Bronx, NY 10461, USA

**Keywords:** mercury, DNA methylation, dopamine, attention, hyperactivity

## Abstract

Attention-deficit hyperactivity disorder (ADHD) is a neurodevelopmental disorder that affects the competence of academic performance and social wellness in children and adults. The causes of ADHD are unclear. Both genetic and environmental factors contribute to the development of ADHD. The behavioral impairments in ADHD are associated with epigenetic changes in genes that are important for neurodevelopment. Among environmental causes of ADHD, the neurotoxin methylmercury (MeHg) is associated with an increased risk for ADHD. Developing children are susceptible to neurotoxic effects of prenatal MeHg exposure. Human epidemiology studies have shown that prenatal MeHg exposure could invoke epigenetic changes in genes that are involved in ADHD. In addition, the pathogenesis of ADHD involves dopaminergic system, which is a target of developmental MeHg exposure. MeHg-induced alterations in the dopaminergic system have a profound impact on behavioral functions in adults. As a trace level of MeHg (around nM) can induce long-lasting behavioral alterations, potential mechanisms of MeHg-induced functional changes in the dopaminergic system may involve epigenetic mechanisms. Here, we review the relevant evidence on developmental MeHg exposures and the risk for ADHD. We also point out research gaps in understanding environmental causes of ADHD.

## Introduction

Attention-deficit hyperactivity disorder (ADHD) is a neurodevelopmental disorder that affects children’s learning ability, social behavior and emotional wellness [1]. Children and adults with ADHD suffer challenges in academic performance, social communication and emotional control. There is no curable treatment for the disease [2]. The symptoms of ADHD include inattention, hyperactivity–impulsivity or both. The estimated incidence of ADHD is 5% in children and 2.5% in adults worldwide [3]. The increased social awareness of ADHD and the great social and economic burden inflicted on the patients call for a better understanding and treatment of the disease [4].

Currently, clinical management of ADHD relies on stimulants including methylphenidate and amphetamine; however, their efficacy is a subject for debate. For example, a recent review pointed out that there is a great uncertainty regarding the effect of long-term treatment with the dopamine agonist amphetamine in adults with ADHD [5]. Furthermore, nearly 10% of patients did not respond to either amphetamine or methylphenidate [6]. In addition, while ADHD mostly afflicts children, many of them show persistent symptoms in adulthood. The causes for ADHD are multifactorial. Both environmental and genetic factors are involved in the development of ADHD [7, 8].

Methylmercury (MeHg) is an organic form of mercury species that naturally occurs in human environment [9]. Human exposure to MeHg comes from eating fish animals that absorb and biomagnify MeHg produced from aquatic microorganisms [10]. The most notable toxic target of MeHg is the brain [11]. Developing fetus is vulnerable to MeHg’s neurotoxicity. As an internal exposure marker of MeHg [12], blood mercury in asymptomatic mothers can cause a long-lasting damage to fetal neurodevelopment [13]. MeHg can form a complex with the amino acid cysteine. The MeHg–cysteine complex is a structural mimicry of the amino acid methionine. Therefore, the complex can take a free pass into the brain through transporters for methionine [14, 15]. MeHg can disrupt cellular redox balance, leading to a cascade of toxic effects [16, 17]. Although there is an uncertainty on the adverse effects of fish eating on the neurobehavioral functions, environmental MeHg exposure can alter DNA methylation levels, an important mechanism in the epigenetic regulation of gene expression [18].

Recent research suggests that environmental MeHg exposure may contribute to the development of ADHD [19]. However, we are far from understanding the causal link between MeHg and ADHD. Herein, we attempt to summarize the current available evidence in support of the pathogenic role of MeHg in the development of ADHD. Although most evidence is indirect, it provides an important base and impetus for future studies. We focused on mechanistic roles of epigenetic effects of MeHg exposure and risk for ADHD, particularly on the modulation of dopaminergic neurotransmission by MeHg. We did not include the heritable effects of MeHg toxicity, genetic susceptibility to MeHg toxicity or significance of latent MeHg effects in age-related diseases, as these subjects were discussed elsewhere [20–23].

### ADHD, Dopamine, and MeHg Toxicity

The development of ADHD involves structural and functional alterations in the developing brain. These alterations are believed to be outcomes of deviation of ‘normal’ brain development [3]. The clinical diagnostic criteria are based on symptoms of learning and social behaviors that are manifested in typical ADHD patients. However, it has to be pointed out that clinical ADHD patients only represent those whose apparent behavior and cognitive developments deviate from ‘normality.’ There is still a proportion of people that exhibit mild and subclinical symptoms of ADHD [24]. The development of ADHD involves multi-systems of neurotransmission in the brain. Dopaminergic neurotransmission plays an important role in the development of ADHD [25].

**Figure 1:**
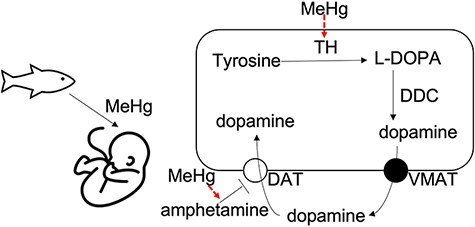
Potential impacts of developmental MeHg exposures on dopamine neurotransmission. The developing brain of fetus is susceptible to environmental exposure to neurotoxins. The primary pathway for dopamine synthesis involves several enzymes including TH and DDC. For the dopamine neurotransmission, MeHg exposure can alter the epigenetic regulation of the TH gene and potentiate the effect of dopamine neurotransmission agonists such as amphetamine [55–58]. TH, tyrosine hydroxylase; L-DOPA, L-3,4-dihydroxyphenylalanine; DDC, DOPA decarboxylase; VMAT, vesicular monoamine transporter 2; DAT, dopamine transporter

Dopaminergic neurotransmission is involved in brain functions including reward system, motor control and emotion regulation [26]. The efficacy of dopamine neurotransmission stimulants in mitigating ADHD suggests that the normal dopaminergic neurotransmission may have been disrupted in ADHD patients [27]. Dopamine synthesis takes several steps, among which tyrosine hydroxylase (TH) is the rate-limiting enzyme to produce dopamine (Fig. 1). Intracellular dopamine is packaged into synaptic vesicles to be released into the synaptic cleft for neurotransmission. Extrasynaptic dopamine levels are regulated and can be transported back into presynaptic neurons by dopamine transporters (DATs).

Studies have shown that the expression of DATs is epigenetically regulated, which is linked to the risk for ADHD [28–30]. For example, in a case–control study on risk factors for ADHD, alterations in DAT-1 expression were linked to ADHD. The study did not find a significant change in overall DNA methylation levels in the promoter region of the *dat-1* gene; however, a possible change in methylation levels in several individual sites of the *dat-1* region was proposed [28]. The importance of *dat-1* epigenetics in the prognosis of ADHD was also demonstrated in a clinical study showing that the methylation status in the promoter region of *dat-1* can predict the treatment outcomes of ADHD with methylphenidate, particularly on oppositional and hyperactive-impulsive symptoms [29]. Furthermore, in a recent investigation on the epigenetics of *dat-1* in ADHD, it is showed that the methylation level in the *dat-1* gene significantly changed in ADHD patients, which was not only related to the severity of ADHD symptoms but also had a predictive value for clinical prognosis [30].

The neurotoxicity of MeHg is mediated via several well-established mechanisms including oxidative stress, mitochondria toxicity and disruption of calcium homeostasis [31–33]. However, it is still unclear whether MeHg exposures at the environmental relevant level invoke the same mechanisms to alter neuronal functions. Recent studies suggest that mechanisms of toxicity induced by the environmental level of MeHg involve epigenetic regulations [34–38], which also play key roles in the transgenerational effects of MeHg [39, 40]. Further, we have recently shown that environmentally relevant exposures of developing human neurons from pluripotent stem cells cause subtle and persistent effects on both neuronal differentiation and neuronal gene expression [41, 42].

MeHg exposure can alter dopamine-mediated neurotransmission [43–50], which can be attributed to MeHg-induced effects on intracellular and mitochondrial calcium regulation [51]. A recent study showed that a dopamine-mediated neurobehavior in *Caenorhabditis elegans* was changed long after cessation of MeHg exposure, suggesting that mechanisms other than calcium signaling are also involved in MeHg-induced alterations in dopaminergic neurotransmission [52]. Given that dopamine-mediated neurobehaviors can be altered by environmental chemicals and the effects are transgenerational [39, 53, 54], studies on the role of epigenetics in MeHg toxicity and its implication for the risk of ADHD are timely and meritorious. In one such *in vitro* study, it was shown that MeHg exposure (1 nM) can repress the expression of TH. The study further investigated methylation status at the promoter region of the TH gene and showed that tri-methylation of histone H3 lysine 27 was significantly increased following MeHg exposure (1 nM) [55]. The importance of dopamine systems in MeHg’s toxicity was corroborated in the *C. elegans* study showing that reduced swimming speeds following MeHg exposure were modulated by the homolog of the TH gene [52]. Furthermore, a recent study showed that the effect of MeHg on neurobehavior functions invoked mechanisms of sperm epimutation, a heritable change in differential DNA methylation regions [39].

The importance of dopamine metabolism in MeHg’s neurotoxicity and its implication in ADHD was also supported by behavioral studies in rodents. A study in female rats showed that their behavioral sensitivity to d-amphetamine was increased following developmental exposure to MeHg [56]. In another study with male rats exposed to MeHg during adolescent development, it was shown that the effects of MeHg exposure on adult neurobehaviors including attention and memory were augmented by the dopamine agonist, d-amphetamine [57]. A follow-up study concluded that adolescence was vulnerable to MeHg and d-amphetamine, and the effect persisted in adulthood [58]. These studies provide important bases for the involvement of dopamine neurotransmission system in behavioral toxicity of MeHg, particularly in the behaviors related to ADHD [57]. However, a direct link between MeHg and ADHD via epigenetic mechanisms remains scarce.

### Environmental MeHg Exposure and ADHD

Developing brains are especially sensitive to MeHg toxicity. Several large cohort studies investigated prenatal and postnatal MeHg exposures and neurodevelopmental outcomes in children. In the Seychelles Child Development Study, although the study did not reveal any significant adverse associations between MeHg exposure and a series of neurobehavioral outcomes [59], significant adverse associations between scholastic achievement and postnatal MeHg exposure were noted particularly in males [60]. Importantly, the study showed that some measures of neurodevelopmental tests were improved rather than adversely affected. This is in contrast with the conclusion of another large cohort study that showed that developmental MeHg exposure adversely affects neurobehavioral functions including attention, memory and verbal functions [61, 62]. The subtle effects of environmental levels of MeHg on neurobehavioral functions in these studies suggest that nutritional factors and co-exposed neurotoxins in the fish may have compounded functional measures of developing brain [63, 64]. In addition, the integrity of epigenetic regulation in the developing nervous system is extremely susceptible to environmental exposures [65, 66]. The potential role of epigenetic alterations by MeHg exposure may have contributed a significant effect in the observed neurofunctional measures (for more on this, see these reviews on MeHg-induced epigenetic alterations [67–69]).

MeHg exposure has been described as a risk factor for ADHD, given that the developing nervous system is most sensitive to the neurotoxicity of MeHg [70–72]; indeed, several studies have shown that mercury exposure, in the form of thimerosal (a mercury-based vaccine preservative), may be positively related to increased occurrence of behavior phenotype of ADHD [73, 74]. In addition, the association between prenatal MeHg exposure and the risk of ADHD was shown in a prospective cohort study in the Canadian Arctic and other cross-sectional studies [19, 75, 76]. These studies suggested that cord blood mercury was associated with higher scores of attention problems and scores of the Disruptive Behavior Disorders Rating Scale with ADHD. However, another cohort study in New Bedford, MA, reported an opposite conclusion, namely, that low mercury level is associated with ADHD behaviors [77]. The frequency of fish consumption is positively related to body mercury levels [78]. Intriguingly, an inverse relationship between mercury levels and risk for the behaviors of ADHD was shown in the cohort study in New Bedford, MA. This effect is probably modified by the level of fish consumption, which provides nutritional factors that are of benefit to brain development. For instance, the incidence of ADHD was reported to be decreased in groups eating the Mediterranean diet, and fish is an important component of the diet [79]. The apparent inconsistency in the conclusions from these studies may also reflect the inherent difference in the populations, such as diet and genetic variations.

In addition, neuronal differentiation and migration in the developing brain require numerous epigenetic modifications to ensure proper regulation of gene expressions and integrated function [80]. Epigenetic regulations in the developing brain are susceptible to environmental exposures [67]. MeHg exposure at trace levels could induce long-lasting and transgenerational epigenetic effects [39, 40]; however, what is less understood is how environmental MeHg exposure might alter epigenetic regulations in neuronal cells *in vivo* and its significance in behavioral outputs of ADHD. Apparently, a mechanistic understanding of MeHg exposure through eating fish and the risk for ADHD may suffice to generate a new hypothesis for future investigations on epigenetic factors that contribute to environmental influences on brain development [18, 81]. Furthermore, understanding epigenetic mechanisms of MeHg’s toxicity is helpful in identifying vulnerable targets and refining measures of developmental outcomes in human studies [82].

Studies on human epigenetic alterations following MeHg exposure used epigenetic markers in blood cells or saliva to infer possible epigenetic influences of MeHg on the brain [18, 81, 83, 84]. Recent epidemiology studies demonstrate that prenatal exposure to MeHg alters epigenetic markers in several genes that are involved in the regulation of neurodevelopment [18, 85]. For example, in the Nutrition Cohort 2 of the Seychelles Child Development Study, prenatal MeHg exposure was associated with increased levels of DNA methylation at the cytosine of CG dinucleotides located at gene-expression regulation sites [18]. The affected genes include brain-derived neurotropic factor (*BDNF*), glucocorticoid receptor (*NR3C1*) and glutamate receptor subunit NR2B (*GRIN2B*), all of which had been shown to be involved in the development of ADHD in other independent studies [86–88]. Increased methylation levels of *NR3C1* associated with prenatal mercury exposure were also reported in another human study showing that the methylation level of *NR3C1* was increased in those with an average mercury level of 0.17 µg/g compared with the reference mercury level of 0.01 µg/g [85].

It has been recognized that perinatal MeHg exposure adversely affects neurobehavior development [13]. A birth cohort by Maccani *et al*. showed that prenatal mercury exposure increased the risk for poorer quality of movement, poorer self-regulation and increased signs of physiologic stress [89]. In addition, toenail mercury tertiles are associated with 339 CpG loci, with an average methylation difference of >0.125. The study also showed that the prenatal mercury level in the toenails of infants is associated with several genes with altered methylation levels, which include transcription elongation regulator 1-like (*TCERG1L*), a possible factor involved in ADHD [90]. Because significant changes in the methylation level of many other genes were also noted in the study, it is difficult to conclude the alteration of methylation level in *TCERG1L* is a direct effect of mercury.

A recent new study carried out in Spain revealed that postnatal mercury exposure was associated with an increased risk for ADHD. The study also showed that boys were more vulnerable than girls to these effects [91]. The study further demonstrated that the polymorphism in *BDNF* modified the association between mercury and behaviors of ADHD. The sex-specific effect on DNA methylation levels following prenatal mercury exposure was also reported in a cohort study in Japan [92]. Specifically, the study showed that hyper-methylation in one locus of the gene of haloacid dehalogenase-like hydrolase domain-containing protein 1 (*HDHD1*) within the transcriptional regulation site was only noted in boys. Furthermore, the temporal changes of epigenetic effects related to prenatal mercury exposure were shown in a study on prenatal mercury exposure and neurocognitive effects [93]. The study further showed that alterations in DNA methylation levels induced by prenatal mercury exposure varied from early to mid-childhood, suggesting that the interaction between mercury and DNA methylation regulation may have been compounded by other factors during development and that the observed changes were indirectly caused by mercury. Methylation of cytosine at CG dinucleotides can be oxidized to hydroxy-methylation, which can independently regulate gene expression [94]. The DNA hydroxy-methylation level was lower in those with higher prenatal mercury levels, which was attenuated from early childhood to mid-childhood [84]. Another investigation of newborns on global DNA methylation level and prenatal mercury exposure showed that the methylation level in the gene of transcription elongation factor A (SII) N-terminal and central domain containing 2 (*TCEANC2*) was associated with cord blood mercury levels [95]. The *TCEANC2* gene is a known risk factor for sporadic Parkinson’s disease [96]; however, the implication of the epigenetic changes to neurobehavioral functions in developing children is unknown.

As mentioned before, the effects of prenatal mercury exposure on the epigenetic markers can be modulated by nutritional factors as well as other toxins. For example, the association between DNA methylation level and prenatal mercury was modified by *in utero* exposure to arsenic [97]. Another neurotoxin that coexists with MeHg in fish [98], namely polychlorinated biphenyls, also had a significant effect on DNA methylation profiles in blood leukocytes [99]. In addition, nutritional elements also affect global DNA methylation levels. One of the mechanisms of MeHg toxicity is the inhibition of the activity of enzymes requiring selenium as a cofactor [9]. Studies have shown that maternal blood selenium is associated with global DNA methylation levels in both pregnant mothers and newborns [83]. Consequently, the disruption of selenoprotein activity and synthesis by MeHg can interfere with DNA methylation of developing brain. Furthermore, prenatal mercury exposure can induce changes in micro RNA profiles in the placenta and cervix, respectively [100, 101], which may lead to altered regulation of epigenetics during fetal development. Taking together, these studies provide important clues on how developmental MeHg exposure alters brain functions and potential effects on the epigenetic control of genes associated with neurotransmission. Several important questions regarding epigenetic effects of MeHg need to be answered. The first is what is the mechanism of MeHg-induced alterations of the DNA methylation level. Finding a mechanistically trackable DNA methylation marker following MeHg exposure will facilitate the research on biological markers that reflect MeHg toxicity. Secondly, what are the epigenetic programs that modulate the development of dopaminergic neurotransmission. This will help to elucidate the critical developmental window that is vulnerable to the adverse effects of environmental factors such as MeHg. Lastly, as human association studies revealed many DNA methylation loci that can be modified MeHg exposure and exhibit a sex-specific pattern, what is the significance of the epigenic alterations induced by MeHg in behavioral outputs. The recent study on the epigenetic effects of MeHg and neurobehavior outcomes in the model organism zebrafish provides an important base for the understanding of these questions [39].

## Conclusion

ADHD is one of the most common neurodevelopmental disorders. Although the pathogenesis of ADHD is not fully understood, exposure to environmental contaminants is associated with the disease. Current evidence suggests that epigenetic regulatory mechanisms such as DNA methylation are a target of environmental MeHg exposure. The investigations on the link between DNA methylation and prenatal MeHg exposure have shown that MeHg exposure may be associated with the pathogenesis of ADHD. Although MeHg exposure is associated with ADHD, the behavioral impacts of MeHg-induced epigenetic alterations warrant further investigations. Furthermore, MeHg exposure may be associated with the epigenetic regulation of genes involved in dopamine metabolism. Developmental MeHg exposure alters the pharmacological effects of dopamine agonists through the interaction with dopaminergic system. These studies provide important clues on how developmental MeHg exposure alters brain functions and its effects on the epigenetic control of genes associated with dopaminergic neurotransmission. However, further studies on the role of MeHg exposure in epigenetic alterations are needed to better understand the association between MeHg and ADHD.

The multifactorial nature of the causes for ADHD suggests that MeHg exposure can significantly alter neurobehavioral outcomes in animal models. As epigenetic marker is particularly susceptible to environmental factors, further investigations on the epigenetic effects of MeHg will shed new insights into the mechanisms of environmental causes of ADHD.
